# Metapresence: a tool for accurate species detection in metagenomics based on the genome-wide distribution of mapping reads

**DOI:** 10.1128/msystems.00213-24

**Published:** 2024-07-09

**Authors:** Davide Sanguineti, Guido Zampieri, Laura Treu, Stefano Campanaro

**Affiliations:** 1 Department of Biology, University of Padova, Padova, Italy; Boston College, Chestnut Hill, Massachusetts, USA

**Keywords:** metagenomics, species identification, read distribution, coverage evenness

## Abstract

Shotgun metagenomics allows comprehensive sampling of the genomic information of microbes in a given environment and is a tool of choice for studying complex microbial systems. Mapping sequencing reads against a set of reference or metagenome-assembled genomes is in principle a simple and powerful approach to define the species-level composition of the microbial community under investigation. However, despite the widespread use of this approach, there is no established way to properly interpret the alignment results, with arbitrary relative abundance thresholds being routinely used to discriminate between present and absent species. Such an approach can be affected by significant biases, especially in the identification of rare species. Therefore, it is important to develop new metrics to overcome these biases. Here, we present Metapresence, a new tool to perform reliable identification of the species in metagenomic samples based on the distribution of mapped reads on the reference genomes. The analysis is based on two metrics describing the breadth of coverage and the genomic distance between consecutive reads. We demonstrate the high precision and wide applicability of the tool using data from various synthetic communities, a real mock community, and the gut microbiome of healthy individuals and antibiotic-associated-diarrhea patients. Overall, our results suggest that the proposed approach has a robust performance in hard-to-analyze microbial communities containing contaminated or closely related genomes in low abundance.

**IMPORTANCE:**

Despite the prevalent use of genome-centric alignment-based methods to characterize microbial community composition, there lacks a standardized approach for accurately identifying the species within a sample. Currently, arbitrary relative abundance thresholds are commonly employed for this purpose. However, due to the inherent complexity of genome structure and biases associated with genome-centric approaches, this practice tends to be imprecise. Notably, it introduces significant biases, particularly in the identification of rare species. The method presented here addresses these limitations and contributes significantly to overcoming inaccuracies in precisely defining community composition, especially when dealing with rare members.

## INTRODUCTION

In the last few decades, high-throughput sequencing technologies have boosted our comprehension of microbial communities, such as those present in the human gut (1, 2), in natural environments (3–5), and those relevant for biotechnological applications (6). The most widely used sequencing approaches for microbiome research are metataxonomics and metagenomics (7). Metataxonomics consists of the targeted sequencing of marker genes, usually focusing on hypervariable regions present in the 16S rRNA gene. This approach allows the clustering of highly similar reads into operational taxonomic units or amplicon sequence variant clusters from which the microbiome composition and diversity can be derived (7–9). By contrast, metagenomics relies on shotgun sequencing of the total DNA extracted from a microbial sample. Its popularity derived from its ability to not only delineate community composition and diversity but also to functionally characterize the microbes present in the sample (7). Moreover, different studies have highlighted the higher taxonomic resolution and precision of shotgun DNA sequencing as compared to 16S amplicon sequencing (7, 10). Notably, it was demonstrated that metagenomic approaches can go beyond the species level allowing the investigation of single strains present in a microbiome (11–13).

Different approaches allow for defining the microbial composition of a given sample starting from shotgun metagenomic sequencing data. In a typical genome-centric workflow, metagenome-assembled genomes (MAGs) are reconstructed by assembling the sequencing reads and clustering the obtained contigs (14). Sometimes, the sequencing coverage of the low-abundant organisms might be insufficient to support a *de novo* assembly (14); however, assembly-free profiling methods allow to mitigate this problem. Among these methods, marker gene-based tools, such as MetaPhlAn, allow profiling microbial communities by mapping reads against sets of clade-specific marker genes, potentially achieving species or strain-level resolution (15). Despite being potentially very powerful, this approach can classify only a small fraction of sequences in a metagenomic sample, preventing more detailed analyses (16).

Alternatively, assembly-free profiling can be used to classify shotgun reads from microbial samples using a set of publicly available genomes or MAGs as reference and specific indexing schemes for the respective sequence database (17). This approach is becoming established since the number of MAGs is fastly growing and they often represent a significant fraction of microbial diversity in well-studied environments (16). Sequencing reads of each sample of interest can be aligned against the available genomes to define the sample-specific microbial community composition and the relative abundance of the associated taxa (6, 18).

With each MAG potentially representing a microbial species, alignment-based approaches can be used to infer the microbial composition and the species abundance. There are different tools that can convert the BAM files obtained from the reads assembly into coverage values or relative abundance, including checkM (19) and coverM (20). For the sake of having a clear representation of the microbial composition in a sample, however, it is necessary to apply a rationale to discriminate between present and absent species. This apparently trivial problem is made complex by the fact that homologous sequences, horizontal gene transfer events, prophage sequences, and even misassemblies can cause an incorrect assignment of reads to genomes, potentially resulting in the erroneous identification of species in the sample under investigation (21). Several metagenomic studies use coverage values of the reference genomes, or other parameters like the number of mapped reads, to calculate relative abundance and to call species as present or absent based on arbitrary thresholds (6, 21). However, since reads can be mismapped, this approach can lead to a high number of false positives, or an overestimation of the number of species present when setting a low threshold of relative abundance. Conversely, a higher threshold may cause the exclusion of low-abundant species, especially for complex microbial communities (21).

Metrics such as coverage, and the directly derived relative abundance, are genome-wide averages, which cannot be used to assess the precise genomic distribution of the mapped reads. This limitation hinders the discrimination between genomes having homogeneous coverage, and those with reads mapped on specific regions only (22). A highly uneven coverage is usually representative of species not present in the sample, but sharing genomic regions with others. The use of relative abundance alone can thus result in an erroneous species identification. The use of additional criteria such as the evenness of the mapped reads on a genome represents a more accurate criterion to assess their presence in the sample. As already suggested by Olm and colleagues (22), the sequencing breadth—the fraction of the genome covered by at least one read—can be used for this purpose. By comparing the real breadth with its expected value at a given coverage, it is possible to infer whether the reads span on the entire genome or cover only specific regions—the latter being representative of a lower sequencing breadth than expected.

In the present study, we investigated the use of different metrics for the identification of the species present in a metagenomic sample, confirming the assessment of mapped read distribution as an accurate approach to discriminating them from false positives. Specifically, we tested the ratio of the coverage breadth to its expected value as a metric to evaluate such a distribution, along with a newly developed metric based on the distance between consecutive mapped reads. Through multiple experiments on synthetic and real data, we showed that the comparison between breadth and expected breadth is not a reliable discriminating criterion at low coverages, while consecutive read distance is more robust. By combining the two metrics, we demonstrated that truly present species can be detected down to as few as tens of reads mapping on a microbial genome.

We included our approach into a freely available Python tool—Metapresence—allowing an easy and reproducible calculation of the two metrics starting from a set of genomes and an alignment file. Our tool allows a reliable definition of the species composition of a given microbial sample.

## RESULTS

### Definition of the approach

As a natural application of the classic Lander and Waterman’s model (23), a random genome fragmentation in shotgun sequencing is well approximated by a one-dimensional homogeneous Poisson point process. Hence, when shotgun sequencing reads are aligned back on the genome they originated from, their mapping positions can be seen as realizations of a Poisson process. When reads are paired-end, each of the two groups of mates can be represented as two different Poisson processes.

Based on this assumption, the number of times a given genomic position is covered by a read is a random variable that follows a Poisson distribution with mean *C*, with *C* being the average coverage:


$$C\;=\frac{R_{n}\;\cdot \;R_{l}}{G_{l}}$$

where $R_{n}$ is the number of reads, $R_{l}$ their average length, and $G_{l}$ the genome length.

Therefore, at a given *C*, the expected fraction of the genome covered by at least one read, defined as sequencing breadth $B_{e}$, can be calculated with the following equation:


$$B_{e}=1-e^{-0.883\cdot C}$$

The 0.883 value was empirically derived by Olm and colleagues (22). In particular, the authors generated synthetic reads from two microbial genomes, subsampled them to different total read numbers, and aligned them back on the corresponding genome. Finally, they fit a curve to the resulting coverage and breadth data to derive the 0.883 value.

We define the metric Breadth-Expected breadth Ratio (BER) as the ratio of the observed breadth $B_{o}$ to $B_{e}$ :


$$BER=\frac{B_{o}}{B_{e}}$$

If the sequencing reads are generated from all the regions of a genome, when aligned back, they distribute across its entire length, resulting in similar values of observed and expected breadth. On the contrary, when sequencing reads map only to a fraction of the genome, the observed breadth is lower than expected. Thus, if the GC content and other properties influencing the sequencing efficiency are homogeneous on the entire genome, BER should be close to one, and this result is a reliable confirmation of the presence of the genome in the sample.

However, as demonstrated in the next section, BER is not reliable at small values of *C*. In fact, when the average coverage is small, the reads essentially do not overlap, and the value of *B_e_* reflects this condition, since *B_e_* approaches *C* when *C* is small. According to that, if a small number of reads map on large genomic regions, they tend not to overlap. Thus, at low *C* values, BER alone cannot clearly distinguish the case where the reads span the entire genome from a situation where they cover just a specific genomic region. Therefore, to take into account this possibility, we developed a novel metric—Fraction of Unexpected Gaps (FUG)—based on the distance between consecutive non-paired reads.

In a one-dimensional Poisson point process, the inter-point distance follows an exponential distribution. Therefore, the distribution of the distances between the mapping position of consecutive non-paired reads follows an exponential distribution with a parameter λ that can be estimated from the results of the read alignment:


$$\lambda =\frac{R_{n}\;}{G_{l}}$$

Thus, we can define the nearest integer number to the expected distance between two consecutive reads as follows:


$$\Delta =round\left(\frac{G_{l}\;}{R_{n}}\right)\;=round\left(\frac{1}{\lambda }\right)$$

Given that the number of distances between consecutive non-paired reads corresponds to the number of reads within each set of non-paired reads minus one, we define $p_{d}$ as the fraction of observed distances having a value equal to *d*:


$$p_{d}=\frac{N_{d}\;}{R_{n}-1}$$

where $N_{d}$ is the number of observed distances with value *d*.

We therefore define FUG as:


$$FUG\;=\frac{\Delta \;-\;\sum_{d=\Delta }^{G_{l}}\;\;p_{d}\;\cdot \;\left(d\;-\;\Delta \right)\;}{\Delta }$$

FUG is essentially an approximation of the cumulative distribution function of the exponential distribution for X > Δ. Thus, when the reads are uniformly distributed on the genome, the expected value of FUG, ${FUG}_{e}$, is:


$$FUG_{e}=1-e^{\lambda \cdot ∆}=1-e^{\lambda \cdot \frac{1}{\lambda }}\approx 0.632$$

When considering paired-end reads, Metapresence separately calculates the FUG value for two groups of non-paired reads, which are constituted by either all the mates mapping as “first in the genome” (upstream), or those that map “as second” (downstream). For each group of mates, two arbitrary reads are added, one mapping to the first position of the genome, and the other to the last. This artifice is performed since we observed situations where reads map uniformly on genomes not really present, yet only on a single region. The two arbitrary reads are necessary, from an algorithmic point of view, to identify these genomes as absent.

While the exponential distribution is continuous, genomic positions are obviously discrete values. The definition of the expected distance Δ is therefore an approximation since the ratio of the genome length to the number of reads is represented by a real number. When the number of mapped reads is low (Δ is high), this approximation is negligible. However, when the number of mapped reads is high (Δ is low), the FUG values deviate from the expected. As we show in the next section of the work, FUG is not reliable at high coverage values.

**Fig 1:**
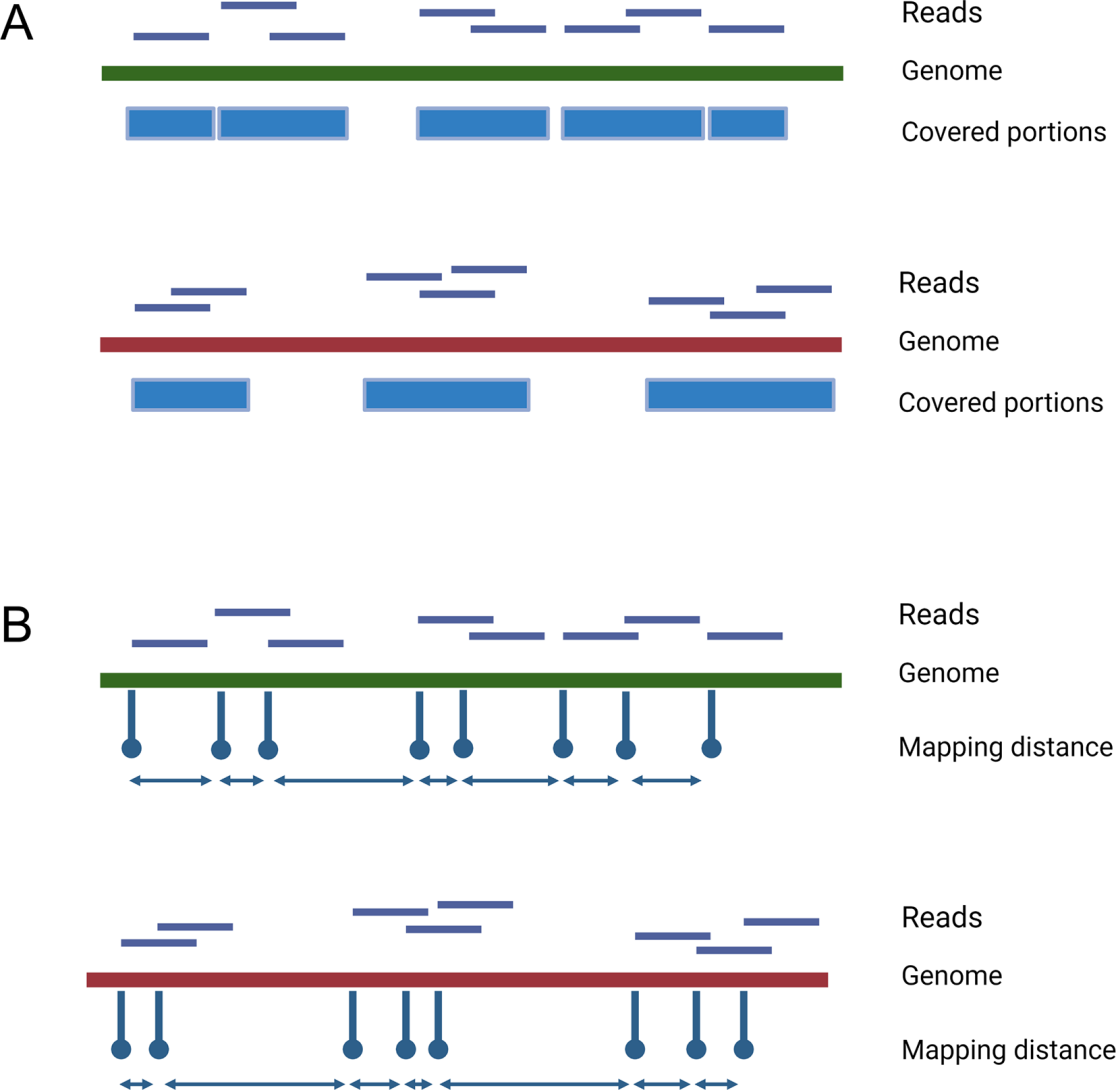
Schematic representation of different read mapping patterns. In both A and B, reads mapping on the green genome spread across its entire length, whereas reads mapping on the red one are confined in one or more delimited regions. (**A**) Although the number of mapping reads is the same, the green genome has a higher breadth of coverage than the red one. (**B**) The bidirectional arrows graphically show the distance between consecutive reads, which is quantified as the distance between initial read positions, depicted as hairpins. Large gaps between consecutive reads are more frequent for the red genome.

A schematic representation of the various mapping patterns that the metrics can distinguish is reported in Fig. 1. In all the following analyses, we set a detection limit of 80 mapped reads. This limit is necessary to ensure a minimum number of reads per group of mates for an accurate approximation of the distance distribution, and, consequently, a reliable calculation of FUG. Thus, all the genomes with fewer than 80 mapped reads are, by default, considered absent.

### BER and FUG effectively distinguish between present and absent species in a synthetic microbial community

To test the performances of BER and FUG metrics in the identification of species present in metagenomic samples, we used the Critical Assessment of Metagenome Interpretation (CAMI) (24) data set as a first trial. This medium-complexity data set is composed of two different sets of synthetic reads generated from 132 microbial genomes, here defined as “sample 1” and “sample 2.”

To evaluate the effectiveness of BER and FUG metrics to correctly identify the genomes within a pool of similar ones, we expanded the data set of 132 original genomes by introducing an additional 170 genomes that were not included in the read generation step. These additional genomes were selected so as to have an Average Nucleotide Identity (ANI) between 80% and 95% with at least one of the 132 original ones.

After the alignment of the synthetic reads to the database generated, we used Metapresence to calculate, for each genome, the FUG and the BER values. We defined as “present” only the genomes with BER and FUG values, respectively, higher than 0.8 and 0.5. The two FUG values were calculated independently for the two groups of mates. These thresholds, determined through various analyses, have proven to be effective in distinguishing between present and absent species (Fig. S1). However, the choice of higher or lower thresholds can be tailored to specific stringency requirements. As mentioned above, all the genomes under the established detection threshold of 80 reads were considered as absent.

**Fig 2:**
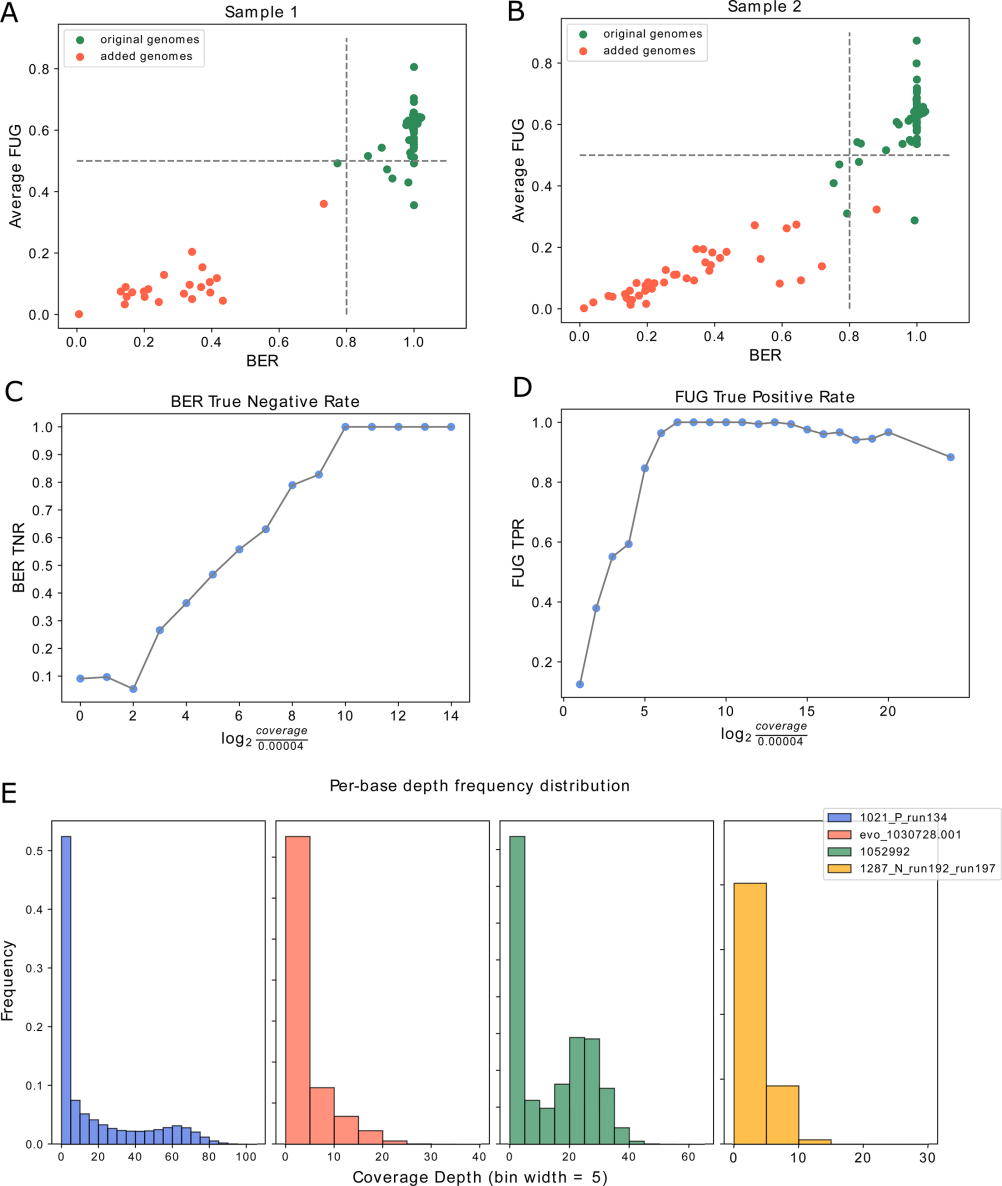
Performances of the metrics on the CAMI data set. (**A and B**) Scatterplots of BER values and the average of the Fraction of Unexpected Gaps (FUG) values for both groups of mate sequences and considering all the genomes with more than 80 mapped reads in sample 1 (**A**) and sample 2 (**B**). Green dots correspond to the genomes of the CAMI data set, while red dots to those added as negative controls. The gray dashed lines represent the suggested thresholds. (**C and D**) Association between the TNR of BER (**C**) or the TPR of FUG (**D**) and the coverage of a specific genome. On the x-axis are reported the log_2_ values calculated for the coverage. TNR and TPR at any given x value are calculated on all the genomes with coverage value comprised between $0.00004\cdot 2^{x-1}$ and $0.00004\cdot 2^{x}$, with x ∈ N. (**E**) The frequency distribution of the per-base sequencing depth of the genomes included as false negatives on the CAMI medium-complexity data set. In the figure legend, the name of each genome is reported. The distribution highlighted in yellow in the figure is obtained from the corresponding genome in sample 2, while the others are from the corresponding genomes in sample 1.

In sample 1, out of the 170 added genomes, only 22 were above the detection threshold, and none was considered as present based on the combined values of BER and FUG metrics (Fig. 2). This outcome resulted in a True Negative Rate (TNR) of 1. Similarly, for sample 2, none of the 40 added genomes above the detection threshold was considered present, resulting in a TNR of 1. In both sample 1 and sample 2, 127 out of 132 original CAMI genomes were correctly identified as present, resulting in a True Positive Rate (TPR) of 0.96. Figure 2A and B illustrates the metric-based separation between the original and the added genomes in sample 1 and sample 2, respectively.

To verify the correlation between the precision of the two metrics and the genome coverage, we systematically downsampled the mapped reads eight times for both sample 1 and sample 2. The downsampling continued until reaching a fraction of 0.0001 of the original number of mapped reads, reducing the count from approximately 50 million to around 5,000 mapped reads. We grouped the genomes in bins according to their coverage and we calculated TPR and TNR of FUG and BER for each bin (see Methods, Evaluation of TPR and TNR at different coverage values).

By applying the aforementioned thresholds, the TPR of BER and the TNR of FUG remained constant regardless of the coverage. As expected, the TNR of BER strongly decreased when the number of reads mapped on a genome was low (Fig. 2C). Meanwhile, the TPR of FUG decreased as the coverage value exceeded 1 (Fig. 2D).

Therefore, we recalculated TPR and TNR for samples 1 and 2 using only BER to assess the presence of genomes with coverage higher than 1, and both BER and FUG for genomes with lower coverage. While the TNR remained equal to 1 for both samples, in sample 1 the calculated TPR was 0.99, while in sample 2 the TPR was 0.98. Therefore, using FUG to assess the presence only of low-coverage genomes increased the precision of the method. These results evidenced that the use of BER and FUG is complementary in the evaluation of present and absent species in a synthetic shotgun sequencing data set.

Focusing on the four false-negative genomes in both samples 1 and 2, we hypothesized that the low values of the metrics might be attributed to an uneven distribution of the mapped reads. When a species present in the sample shares genomic regions with one or more other present species, sequencing reads may mismap across their genomes. If, for instance, the reads mismap from a highly abundant species to a low-abundant one, then at least one region of the low-abundant genome may have coverage much higher than the rest of the genome. At an increasing mismapping rate, coverage distributions can thus display altered properties reflecting a biased read assignment. Figure 2E shows the distribution of the per-base depth for each false negative. These tailed or bimodal distributions are indicative of the presence of one or more genomic regions having an average coverage higher than the value calculated for the whole genome. This arguably led to a distortion of the BER and FUG values, which can be considered as measures of coverage evenness.

### Dependence of BER values on sequence similarity

In the previous section, we evaluated BER and FUG as metrics capable of distinguishing a specific genome (from which sequencing reads are generated) from others that are similar yet distinct. Thus, we assumed that these metrics can quantify the similarity between a given genome and the one actually sequenced. To validate this assumption, we chose to investigate the correlation between BER and sequence similarity by focusing on the NCBI representative genome of *Rickettsia endosymbiont* of *Ceutorhynchus assimilis*. This bacterial species was selected due to the wide range of ANI values calculated in the comparisons with other representative genomes, which span from 75% to 100%. Then, we generated 50 million synthetic paired-end reads from the aforementioned *Rickettsia* genome and we aligned them against the other selected genomes (see details on Methods, BER, and sequence similarity).

**Fig 3:**
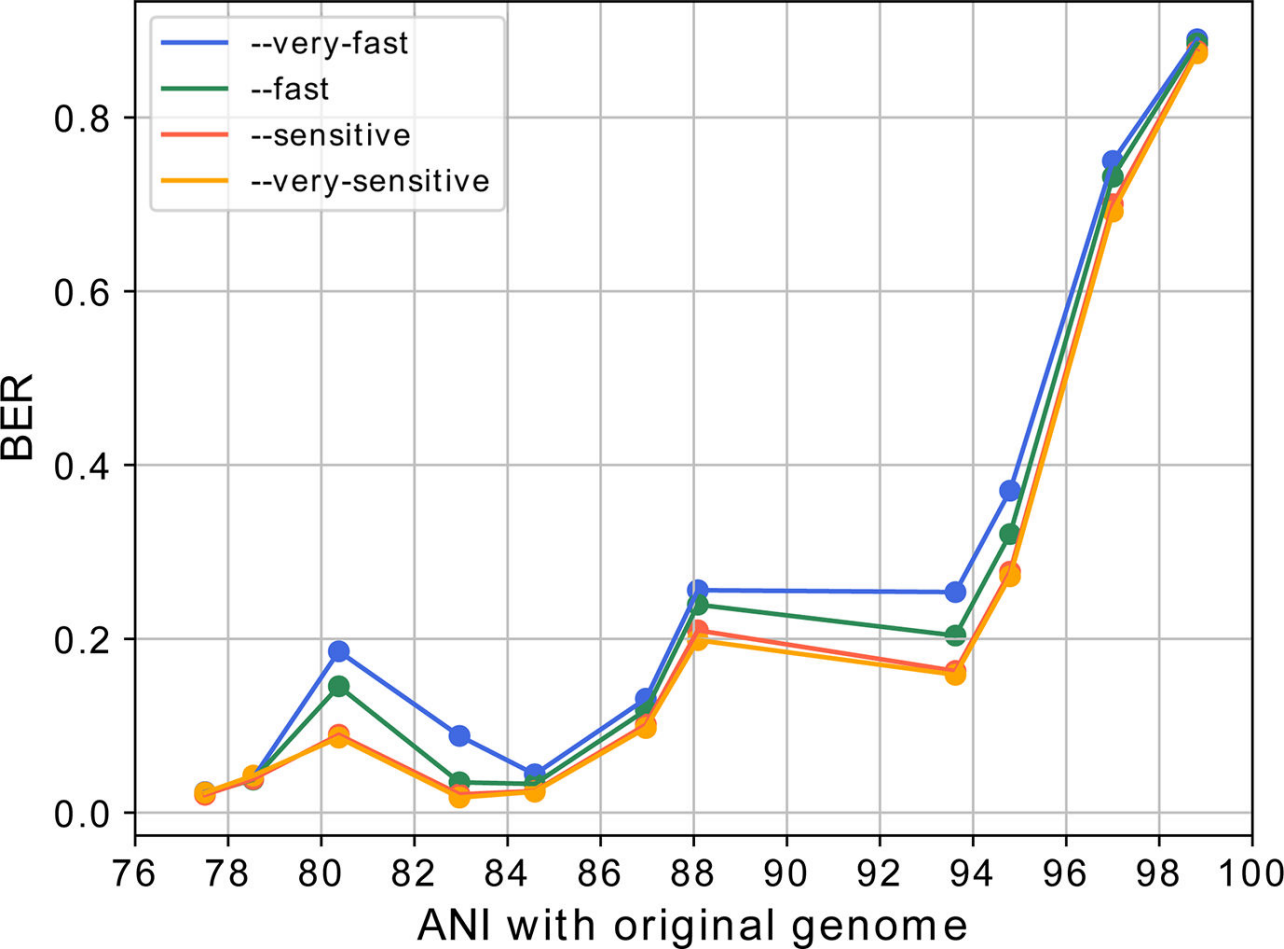
Relationship between BER and sequence similarity. On the x-axis, the average ANI value of *Rickettsia* genomes with respect to the representative *C. assimilis* genome is reported, while on the y-axis the average BER value for the same genomes using different preset modes of Bowtie2 for sequence alignment. For visualization purposes, the genomes in the comparison are grouped into discrete intervals having size 2 based on the ANI values.

Figure 3 shows the relationship between ANI and BER using different preset modes of Bowtie2 for read alignment. As expected, BER values increase proportionally with the similarity of a genome to the one from which the sequencing reads were generated, suggesting that BER serves as a measure of global sequence similarity. As shown in Fig. 3, this result is only partially influenced by the sensitivity of read alignment. Subsequently, we performed again the read alignment process with the default mode of Bowtie2, but this time including also the original *Rickettsia* genome. The BER value calculated for the genome from which the synthetic reads were generated (i.e., ANI = 100%) was 0.997. This high value is indeed expected when reads span the entire genome.

### Benchmarking Metapresence against existing metagenomics profilers

To assess the accuracy of the proposed approach relative to existing metagenomic profilers, we decided to compare Metapresence with YACHT and Metaphlan4. YACHT is a recently published tool for species presence/absence detection (25) implementing a statistical test based on ANI. Metaphlan4, instead, is a widely used tool for taxonomic profiling of metagenomic samples that estimates species presence and abundance based on genome-specific marker gene detection (15).

We selected 395 known species and obtained their genomes from the NCBI database. We then used CAMISIM (26) to simulate 10 microbial community profiles and to generate synthetic sequencing reads from these genomes. In particular, the abundance profiles of the communities were sampled from lognormal distributions with parameters μ = 1 for all the simulations and different σ values. Small values of σ mimic communities with many moderately abundant species, while high values simulate communities with few highly dominant species and many rare ones. In our generated communities, up to 50% of the species had a relative abundance at or below 0.01% (Fig. S2). We then expanded the reference genome data set by adding 821 microbial genomes having ANI between 85% and 95% with at least one of the 395 genomes used to generate the synthetic reads. Finally, we used each tool to define the composition of all the generated samples. The performance was evaluated using the TPR, TNR, and balanced accuracy, that is, the mean between TPR and TNR.

Concerning Metapresence, according to the results obtained with the CAMI medium data set, the genomes with coverage higher than 0.1 were considered as present if they had a BER value higher than 0.8, while the genomes with coverage lower than 0.1 were considered as present with BER values higher than 0.8 and FUG values higher than 0.5. As before, all the genomes with fewer than 80 mapped reads, or with metric values lower than the thresholds, were considered absent.

The reference sketch for YACHT was built using our expanded reference genome data set. For the identification of present species, we tested different significance levels and minimum coverage parameters, and we selected the combination resulting in the highest balanced accuracy across all samples (significance = 0.99, minimum coverage = 0.05). Regarding Metaphlan4, the sequencing reads of each sample were aligned against the marker gene database (October 2022), and the results of the alignments were given as input to Metaphlan4. For each species used to generate the synthetic reads, we manually collected the date of its definition and all the possible synonyms from the List of Prokaryotic names with Standing in Nomenclature (27). For the calculation of the TPR, we excluded all the species defined later than 2021, and, for the remaining ones, we parsed the output of Metaphlan4 searching for all the possible synonyms. We tested different relative abundance thresholds and we selected the one giving the highest balanced accuracy across all samples (0.002%).

In addition, we tested a modified version of Metaphlan4 by applying the proposed coverage evenness metrics. Specifically, we calculated BER and FUG for each marker gene starting from the alignment results. We then integrated the metric values in the Metaphlan4 workflow by modifying the tool so that a marker gene with a BER value lower than 0.7 and a mean FUG value lower than 0.4 was considered to have zero reads mapping on it (see Methods, YACHT and Metaphlan 4). In this case, we did not use any relative abundance threshold.

**Fig 4:**
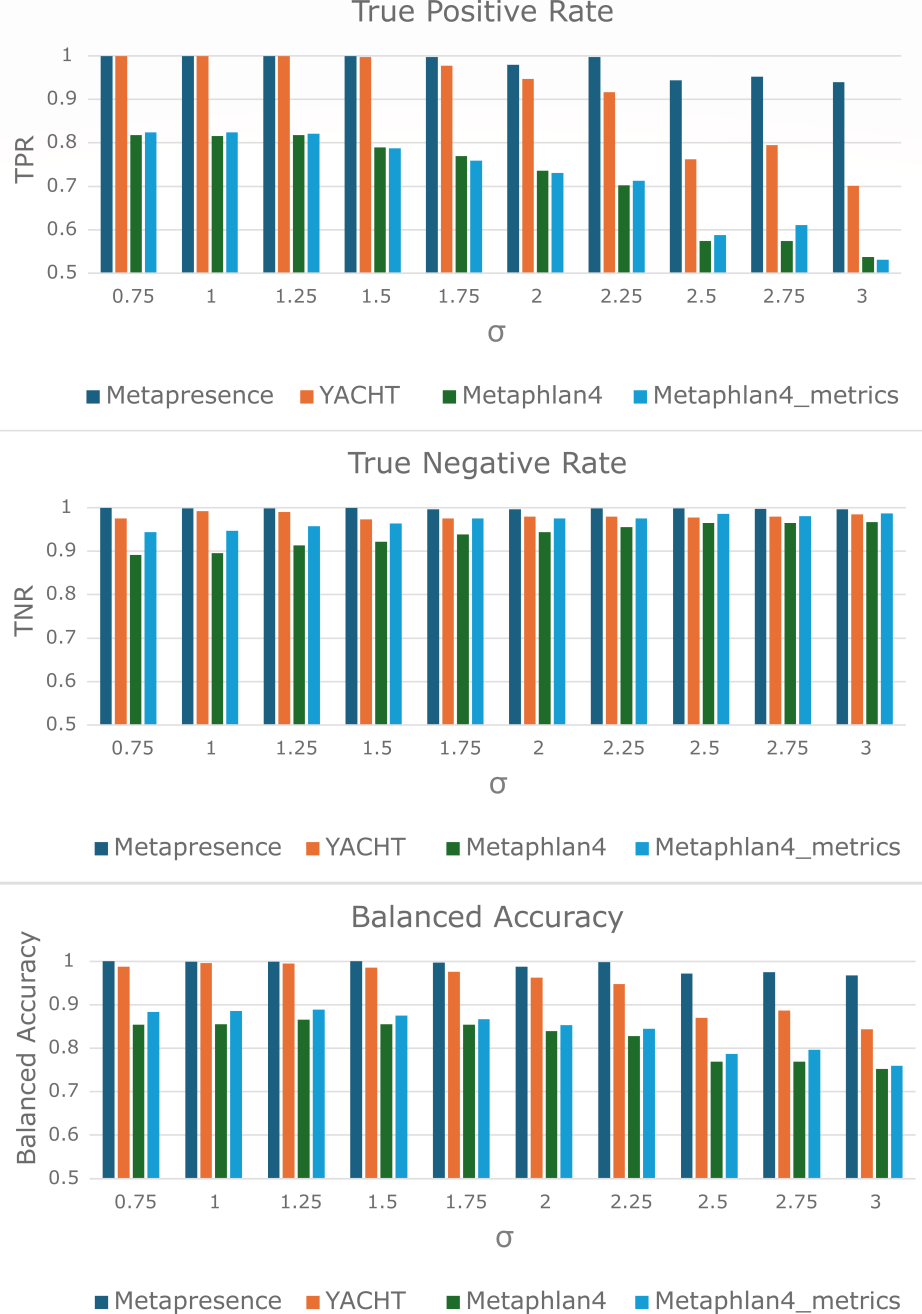
Results of benchmark tests. Each subplot represents the value of the corresponding performance metric for Metapresence, YACHT, Metaphlan4, and Metaphlan4 when used together with the coverage evenness metrics (Metaphlan4_metrics). Each group of bars refers to a synthetic community identified by the σ value of the lognormal distribution used to generate the corresponding abundance profile, as indicated on the x-axis. Depending on the subplot, the y-axis represents the value of true positive rate (TPR), true negative rate (TNR), or balanced accuracy (mean of TPR and TNR) for each tool.

As shown in Fig. 4, Metapresence maintains the highest balanced accuracy across tools in all the test communities, with larger performance advantages at increasing the representation of rare species. Even in communities largely dominated by rare members, TPR and TNR maintained close 0.95 and 1, respectively, suggesting that coverage evenness metrics are robust to heavily polarized coverage distributions. The second best performing tool was YACHT, also characterized by a higher TPR and TNR as compared to Metaphlan4, but displaying lower robustness than Metapresence at high σ values. Interestingly, evaluating the coverage evenness on the marker genes of Metaphlan4 sensibly decreased the number of false positives without impacting TPR.

### BER and FUG precisely identify species in real sequencing data of a mock community

To evaluate how effectively the metrics can discriminate between present and absent genomes with a real read distribution, we downloaded Illumina sequencing data for a mock community composed of 26 bacterial species at different abundance levels (28). For each species, we selected a representative genome among those reported as “complete” in the NCBI database (September 2023). Similarly to the analysis performed using the synthetic reads, we selected 276 genomes among the NCBI representative genomes with an ANI between 80% and 95% with at least one of the 26 genomes putatively present in the mock community.

To simulate a realistic sequencing depth, we performed three rounds of subsampling from an initial pool of over 300 million read pairs, using different randomization seeds to achieve a final data set of 10 million read pairs. For each subsample, we aligned the reads on a reference database obtained from the 302 genomes previously selected and we calculated FUG and BER from the resulting sorted alignment files. The presence or absence of each genome was determined with Metapresence as described in the previous section.

**Fig 5:**
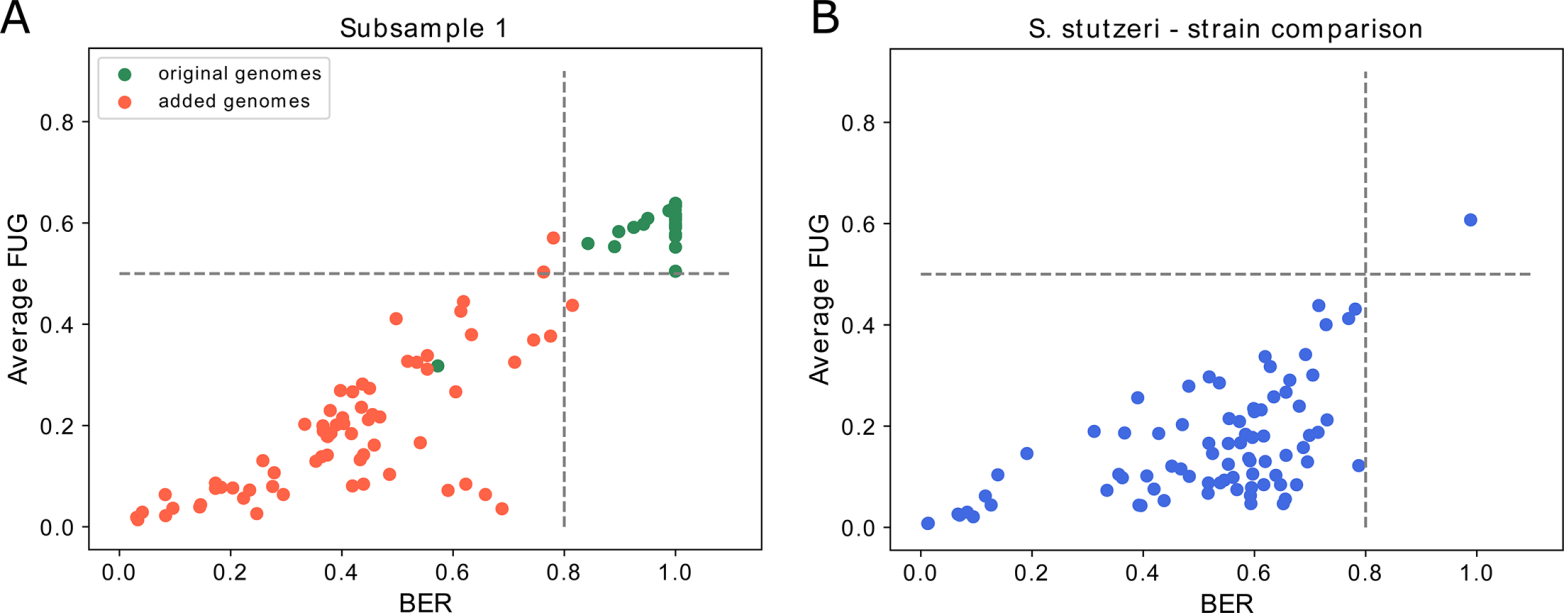
Performances of the metrics on the mock community data set. (**A**) Scatterplots of BER value and the average FUG were values calculated for both the mate reads for the genomes with more than 80 mapped reads in the represented subsample. Green dots correspond to genomes of the species in the mock community, while red dots to those added as negative controls. The gray dashed lines represent the metric thresholds. (**B**) Scatterplot of BER value and the average FUG values were calculated for both groups of mate reads and for all the genomes of *S. stutzeri* downloaded from the NCBI database. The dot with the highest metric values corresponds to the strain *S. stutzeri* RCH2.

Results for one of the subsamples are visualized in Fig. 5A, while they remained essentially identical in all the other subsamples. In all, 24 out of 26 present genomes were identified as present in all subsamples, and all the artificially added genomes were consistently identified as absent. Among the genomes identified as present, the number of mapped reads ranged between 26,000 and 3 million. These results show the high precision of the proposed approach in identifying truly present species in a real shotgun sequencing data set.

To understand in which cases the metrics might fail, an additional investigation was performed on the two false negatives. One genome—the *Nocardiopsis dassonvillei* representative—was misclassified since it was below the detection limit of 80 mapped reads in all the subsamples. The other false negative was the *Pseudomonas stutzeri* representative genome, one of the species used for the generation of the mock data set. A more detailed investigation revealed that *P. stutzeri* was recently included in the newly proposed *Stutzerimonas* genus (29). In addition, *S. stutzeri* represents a particular case of species classification, since it is composed of different “genomovars,” namely phenotypically equivalent genomic groups that can be differentiated using ANI or other equivalent indexes (29). Alignment of the Illumina reads belonging to one subsample on 166 *S*. *stutzeri* genomes downloaded from the NCBI database confirmed that, according to BER and FUG values, only *S. stutzeri* RCH2 was present (BER 0.98, FUG1 0.61, FUG2 0.60), while all the remaining 165 were absent (Fig. 5B).

The comparison of *S. stutzeri* RCH2 with the other *S. stutzeri* genomes revealed an ≈86% average ANI, and a maximum value of 93%, evidencing that RCH2 is clearly distinct from the other strains. We concluded that the sequencing reads were generated from *S. stutzeri* RCH2 (or a highly similar strain) and the false-negative result obtained with Metapresence was simply due to the incorporation of a distantly related strain in the database used for sequence alignment.

### Monitoring the microbiome composition of real-case samples from patients with antibiotic-associated diarrhea

To validate the usage of BER and FUG in a real case study characterized by a complex microbiome such as the human gut, we downloaded sequencing data obtained from fecal samples associated with various clinical studies of *Clostridium difficile* infection (30–32). Data were associated with 63 positive patients and 61 healthy donors. *C. difficile* is recognized for causing antibiotic-associated diarrhea (AAD), a condition characterized by the overgrowth of opportunistic microbes. However, it is noteworthy that less than 30% of AAD cases present a *C. difficile* infection (33). Other bacterial species have been associated with AAD, including *Klebsiella oxytoca, Citrobacter amalonaticus, Clostridium innocuum, Clostridium perfringens, Staphylococcus aureus, Enterococcus faecalis, Enterobacter cloacae,* and *Pseudomonas aeruginosa* (33).

To define the community composition with Metapresence, the human gut database generated by Almeida and colleagues (2) was used as a reference for read alignment. ANI comparison was used to verify that all the AAD-associated species were included in the Almeida et al. database. Hence, the sequencing reads of the clinical studies on *C. difficile* infections were aligned against all the MAGs in the database, and BER and FUG values were calculated, thus defining the community composition.

**Fig 6:**
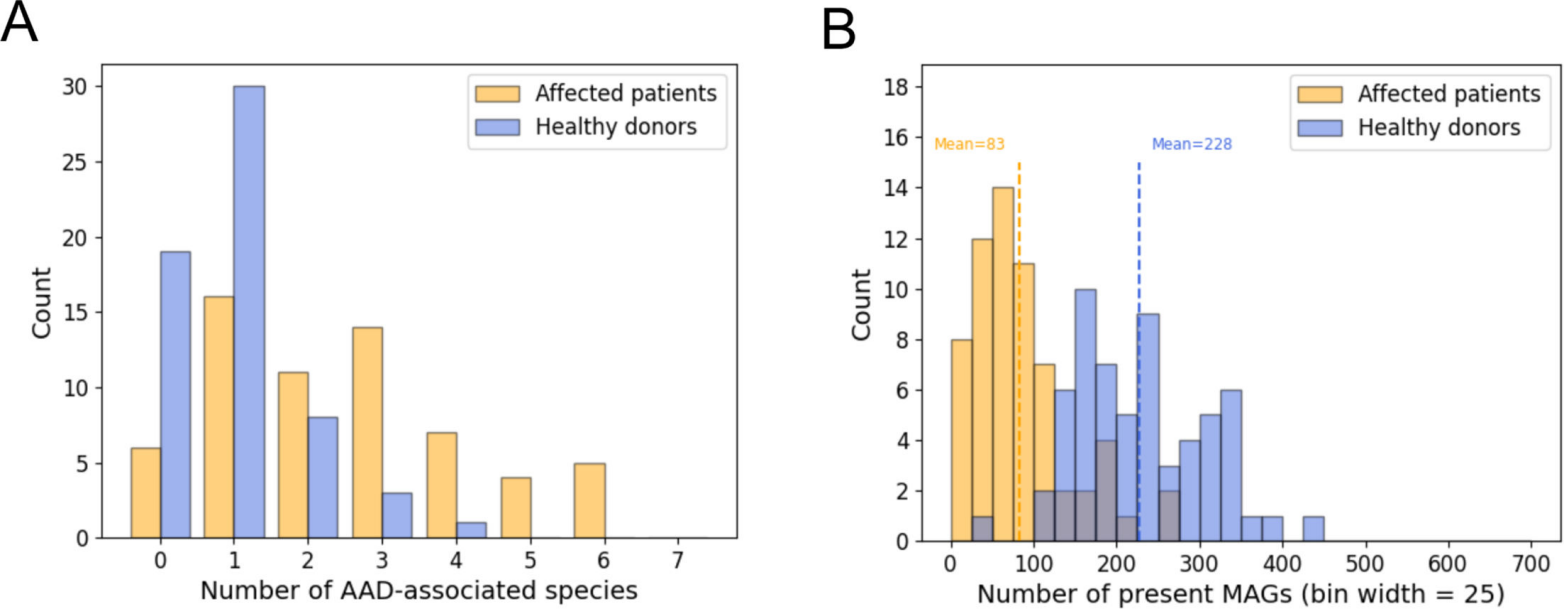
Species identified in AAD-related samples. (**A**) Histogram representing the number of AAD-associated species identified in each sample derived from affected patients or healthy donors. The y-axis represents the number of samples with a given number of AAD-associated species. (**B**) Histogram representing the total number of species identified in samples from affected patients and healthy donors. The y-axis represents the number of samples where the number of species is within a given range.

The number of AAD-associated species identified in patients and healthy donors is reported in Fig. 6A. Approximately 90% of the samples collected from affected patients revealed the presence of one or more AAD-associated species. As expected, these species were identified also in samples from healthy donors, confirming that they are opportunistic pathogens normally inhabiting the human gut (34). However, differential abundance analysis of the pathogenic species between healthy and affected samples (See Methods, Normalized read count metric) revealed that the sum of the read counts in AAD-associated species’ ${(Rc}_{n}$) is higher in affected samples (mean 10.23) than in healthy samples (mean 0.24) (Mann-Whitney U, *P* < 1e-9).

AAD is also associated with gut microbiome dysbiosis, which, like other conditions, is characterized by a reduced microbial diversity in the gut microbiome (35). Hence, we would expect to find a lower number of bacterial species in samples from affected patients. Calculation of BER and FUG revealed that healthy individuals had on average a higher number of species than affected patients (T-test, *P* < 1e-15) (Fig. 6B). This finding is in line with previous knowledge concerning AAD-associated gut microbiome dysbiosis, and it proves that Metapresence can be successfully implemented in the calculation of α-diversity measures of microbial communities.

An additional investigation was performed by focusing the attention on the most abundant MAGs (${Rc}_{n}$ > 1) of the six samples derived from AAD patients where none of the AAD-associated species were identified. In four of these samples (SRR13844386, SRR13844421, SRR13844456, and SRR13844459) we found abundant species belonging to *Klebsiella* genus, such as *K. pneumoniae, K. grimontii,* and *K. variicola*, suggesting that, in this genus, species other than *K. oxytoca* may be associated with AAD. Notably, these six samples were also enriched with species frequently employed in the probiotic management of diarrhea, such as *Bifidobacterium spp*. and *Lactobacillus spp*. (36). This observation suggests that the patients may have been undergoing probiotic treatment.

## DISCUSSION

The importance of this study lies in the need to address the inherent biases associated with utilizing relative abundance thresholds for defining the species-level composition of a metagenomic sample. To address this challenge, the main target was to explore the utilization of mapped read distribution on genomes as a reliable method for discriminating the species present from those absent.

The results obtained from synthetic and mock communities indicate that this approach is highly precise and holds significant potential. Furthermore, the findings from real case studies from AAD samples align with established knowledge (35), suggesting that the proposed method can effectively define the species-level composition of complex microbial communities, such as those found in the human gut.

Comparison with established methods for species identification in metagenomic data revealed that the approach investigated in this work can outperform existing ones, especially in the analysis of communities with a high prevalence of rare species. Interestingly, the integration of the proposed coverage evenness metrics in the Metaphlan4 workflow (15) increased its precision. This result suggests that the evaluation of mapping read distribution may improve the accuracy of methods that rely on read alignment to perform inferences. More thorough analyses are needed to investigate the potential of coverage evenness metrics in test cases going beyond species identification in metagenomic samples.

The most critical point of the proposed approach is the selection of the metric thresholds. According to the tests performed, we defined 0.77 and 0.5 as the optimal thresholds for BER and FUG, respectively. Nevertheless, users have the flexibility to choose a more stringent value for these metrics.

In this work, we have shown that the BER value for a given genome is dependent on the similarity between that genome and the one from which the sequencing reads were obtained. The test performed for the identification of the *Rickettsia endosymbiont of Ceutorhynchus assimilis* revealed that, with a 0.8 BER threshold, Metapresence requires an ANI exceeding 98% between the genome used for read generation and the target genome for alignment. Under this assumption, if reads are generated from strains with an ANI ranging between 95% and 97%, successful species identification using Metapresence may not be achievable unless more permissive thresholds are chosen. More comprehensive analyses can be performed to identify the most suitable values in application scenarios not considered here, taking into consideration the complexity of real microbial communities. Nevertheless, the results of our investigation revealed that, in general, the proposed thresholds can precisely define the species-level composition of microbial samples having different origins.

Given the TNR observed in the analyses performed on synthetic and mock communities, the described approach can improve the outcomes beyond those achieved solely with relative abundance thresholds. In fact, irrespective of the coverage value (or the relative abundance), high BER and FUG values for a genome representative of a given species can reinforce the conclusion that the species is indeed present in a sample. Hence, the proposed method is more suitable than using relative abundance thresholds and particularly for the identification of rare species and those with moderate occurrence.

Moreover, given that higher values of the metrics correspond to increased sequence similarity, we hypothesize that the described approach may also be used in a reliable way for defining the strain-level composition of metagenomes. This could be achieved by employing fine-tuned BER and FUG thresholds for distinguishing genomes representing different strains of the same species within the same sample.

In general, FUG can be calculated through the theoretical and practical description that we have included in this manuscript, and BER value can be readily calculated starting from the output of tools such as inStrain (22) and coverM (20). However, to facilitate the calculation of BER and FUG we developed the Python tool Metapresence.

Overall, we demonstrated the potential of using the distribution of mapped reads on genomes as a criterion to define the presence of species in a metagenomic sample and we envision further development of this approach and its implementation in metagenomic pipelines.

## MATERIALS AND METHODS

### BER and FUG calculation

To calculate the BER metric, it is necessary to calculate the coverage breadth and the average coverage, which are derived from the per-base sequencing depth. More in detail, for a given contig, to calculate the depth at each position, Metapresence generates an array as long as the length of the contig, where each position of the contig is represented by the value in the array with an index equal to that position. All the values are initially set to 0. If a read maps on a given position, +1 is added to the corresponding value in the array, while −1 is added to the value of the array corresponding to the mapping position plus the read length. Multimappers, cigar operations, Phred quality scores, and alignment scores are ignored. Finally, sequencing depth at each position is calculated as the sum of all values in the array preceding the position. For a given genome, the average coverage is given by the average depth across all positions of each contig, while the breadth is given as one minus the ratio of the number of positions with zero depth to the genome length.

The BER metric is given by the ratio of the calculated breadth to the expected breadth, where the latter is obtained using the formula mentioned in the first section of the results.

To calculate the FUG metric, all the contigs of a given genome are arbitrarily joined together in one contiguous sequence. The mapping positions on all the contigs of a given genome are stored in an array. If the reads are paired-end, two arrays are generated: one for the first mates encountered in the sorted bam file, the other for the second. For each contig, the values that are stored are given by the mapping position on the contig plus the sum of the lengths of all the contigs preceding it in the arbitrarily generated contiguous sequence. One arbitrary read is added at the starting position of the contiguous sequence, and one at the ending position minus the average read length.

For each array, the expected distance is calculated as the ratio of genome length to the number of reads in the array, and the FUG metric for each group of mates is then calculated as described in the first section of the results. The artifice of joining the contigs together is necessary for calculating FUG on low-coverage and fragmented genomes. Analyses not included in this manuscript have shown that this approach does not bias the FUG value. Furthermore, the addition of one read at each end of the contiguous sequence not only avoids biasing the FUG value but is also essential in situations where reads uniformly map to a single contiguous region of the genome.

The calculation of the two metrics is implemented in Metapresence. Computational time and resource requirements were measured relatively to different data set scales. This analysis is shown in Fig. S3. When only one process is used, results show that, even when the number of reads is higher than one hundred million and the total size of the reference database is on the order of billions of bases, time consumption remains on the order of minutes and peak memory on the order of a few gigabytes. Parallelization significantly speeds up the computation, increasing however memory consumption.

### Synthetic test data sets

To test the metrics on synthetic data, the Critical Assessment of Metagenome Interpretation (CAMI) medium complexity data set was used (24). This consists of 132 bacterial and archeal genome sequences and 100 bacterial plasmids, together with almost one hundred million 150 bp paired-end reads in fastq format, synthetically generated from the 232 sequences mentioned above assuming an Illumina HighSeq error profile. In particular, all the analyses were performed using the two sets of paired-end reads with an insert size of 270 nucleotides. This data set was chosen since it simulates a microbial community with a known species composition.

To evaluate the effect of read mismapping and to test the sensitivity and specificity of the metrics, the data set was expanded by adding 170 genomes selected among the bacterial representative genomes in the microbial genome NCBI database, and with an average nucleotide identity (ANI) between 0.80 and 0.95 with at least one of the CAMI genomes. The NCBI sequence identifier for each one of these genomes is reported in Table S1, section 1. ANI was calculated using the fastANI software with default options (37), and by setting the CAMI genomes as references and the representative genomes as queries. The plasmid sequences were not included in the alignment step. The reads were aligned using Bowtie2 with default options (38).

To generate more complex synthetic communities, we used CAMISIM (26), giving it as input 395 genomes. These genomes were species-specific and, according to fastANI (37), they had ANI values lower than 95%. We set the genomes_total and num_real_genomes parameters to 395, while a specified log_sigma parameter was used to define ten distinct communities. In particular, we used the following values: 0.75, 1.0, 1.25, 1.5, 1.75, 2.0, 2.25, 2.5, 2.75, and 3.0. For each simulated community, 50 million pairs of reads were generated. All other options were kept to default.

To expand the reference genome data set, we downloaded from NCBI 821 genomes with ANI values between 85% and 95% with at least one genome among those used to generate the synthetic reads. Information about the species forming the synthetic communities and the corresponding sequence identifiers is shown in Table S1, section 5.

### Evaluation of TPR and TNR at different coverage values

To evaluate the potential influence exerted by the coverage on a given genome on the precision of BER and FUG metrics, we downsampled the mapped reads of both CAMI medium’s sample 1 and sample 2 using the option *-s* of *samtools view* (39) and gave as input the sorted bam file obtained from each sample. The subsampling was performed at the following fractions: 0.5, 0.1, 0.05, 0.01, 0.005, 0.001, 0.0005, 0.0001.

We separated original and artificially introduced genomes in two distinct groups, and we clustered them into bins depending on their coverage. Since the coverage value ranged between 10^-3^ and 10^2^ for the original genomes, bin boundaries were defined over logarithmic intervals as follows. For TPR, we clustered all original genomes in all subsamples in bins. Each genome, if detectable in multiple subsamples, could appear in multiple bins depending on the coverage value. The boundaries of the i-th bin were thus given by the following function:


$$B_{\left(i\right),low}=0.00004\cdot 2^{i};\;B_{\left(i\right),up}=0.00004\cdot 2^{i+1}$$

where $B_{(i),low}$ is the lower boundary, $B_{(i),up}$ is the upper one, and 0≤i≤21|iϵN.

The TPR calculation was performed independently for the genomes of each bin, and the result was plotted using a logarithmic scale along the x-axis to facilitate visualization.

For the TNR calculation, the artificially introduced genomes were used. The procedure was identical, with the exception that in this case 0≤i≤14|iϵN.

### BER and sequence similarity

The sequencing reads were generated from the RefSeq genomic sequence of *Rickettsia endosymbiont of Ceutorhynchus assimilis* (GCF_918308855.1_Rickettsia_endosymbiont_of_Ceutorhynchus_assimilis_genomic.fna) with the ART software (40). In particular, the linux-64 version of ART MountRainier (version: 2016–06-05) was used with the following options:

-ss HS25, -p, -l 150, -m 1000, -s 50, -c 50000000.

The genomes included in the database for read alignment were selected taking into account ANI as follows. The ANI values were calculated using fastANI (37) and all the representative genomes in the NCBI database belonging to the *Rickettsia endosymbiont of Ceutorhynchus assimilis*, the *Wolbachia* genus, and the *Rickettsia* genus. We clustered genomes according to the ANI value with the representative *Rickettsia* genome in bins of length 2 from 76 to 100 (i.e., 76–78, 78–80, …, 98–100). For each bin, we randomly selected three genomes except for the 90–92 bin which did not have any genome, and the 98–100 bin which had only two genomes, both selected. The sequencing reads were aligned against the selected genomes, excluding the original *Rickettsia* genome. An alignment was performed for each end-to-end preset mode of Bowtie2, and, for each alignment, we plotted the mean ANI value and the mean BER value for the three (or the two) genomes in each bin.

For the genomes used in the analysis, the NCBI genome sequence identifiers and the corresponding ANI values are reported in Table S1, section 4.

### Benchmark tools

The reference sketch for YACHT (version 1.2.2) (25) was built using the “yacht sketch ref” command, using as input our expanded CAMISIM reference genome data set, comprising the 395 genomes used to generate the synthetic reads and the 821 added genomes. The following options were used: --kmer 31 --scaled 1,000. All other options were kept to default. The sketch for the sequencing reads from each synthetic community was built using the command “yacht sketch ref” and the following options: --kmer 31 --scaled 1,000. All other options were kept to default. For the command “yacht train,” we used an ANI threshold (--ani_thresh) of 0.975, which was the lowest value avoiding the merging of multiple genomes used for generating the sequencing reads. For the command “yacht run,” we tested multiple values with the --min_coverage_list option and we selected the min_cov value resulting in the highest balanced accuracy across samples (min_cov = 0.05), that is, the mean between TPR and TNR. Moreover, we tested both 0.99 and 0.95 as significance thresholds, selecting 0.99 as the one giving more accurate results.

Concerning Metaphlan4 (version 4.0.6) (15), the sequencing reads were aligned against the Metaphlan database (version: October 2022) using Bowtie2 with the following options: --sam-no-hd --sam-no-sq --no-unal --sensitive-local. Metaphlan4 was launched with the option “--input_type sam.” All other options were kept to default. From the Metaphlan4 output, we considered “present” in the sample all the species with relative abundance above a given threshold. Several thresholds in the range of 0%–0.1% were tested, and the one resulting in the highest balanced accuracy was selected (0.002%). To calculate the TPR, we did not take into account all the species used to generate the synthetic communities which were defined later than 2021 according to the List of Prokaryotic Names with Standing in Nomenclature (27), and in particular, to the web version of the database and the “Name” section of the page of each species. Moreover, we searched in the Metaphlan4 output all the synonyms of each species that are indicated in the same database. A true positive was considered present in the sample if at least one of its synonyms was identified in the Metphlan4 output. The number of false positives was calculated by counting the number of species-level genome bins identified in the Metaphlan4 output and above the relative abundance threshold not corresponding to species used to generate the sequencing reads. The TNR was obtained by dividing this number by the total number of species-level genome bins in the database according to the documentation of the tool, that is 26,970.

Concerning the integration of FUG and BER metrics with the Metaphlan4 workflow, from the alignments performed against the Metaphlan4 database we used Metapresence to calculate BER and FUG values for each marker gene present in the database. We modified the Python script of Metaphlan4 (version 4.0.6) by changing the function “map2bbh”—defined at lines 835–895—so that all marker genes with BER value smaller than 0.7 and FUG value (average of the two mate groups) smaller than 0.4 were considered to have zero reads mapping on them. We chose more flexible values of the metrics to account for the small size of the marker genes. The definition of TPR and TNR was performed from the output of the modified Metaphlan4 as described above, and without setting any relative abundance threshold.

### Mock community data set

To test the metrics on real shotgun sequencing data, the mock community defined by Singer and colleagues (28) was used. The sequencing reads were subsampled to three groups of 10 million read pairs, ensuring each pair could appear in only one subsample. The NCBI sequence identifier of the genomes selected as representative for each species of the mock community is shown in Table S1, section 2.

The expansion of the data set with new genomes and the read alignment was performed in the same manner used for the synthetic data sets (Methods, Synthetic test data set). The NCBI sequence identifier of the added genomes is shown in Table S1, section 2.

### AAD data set

The sequencing reads of samples derived from 63 AAD-affected patients and 61 healthy donors were recovered from multiple studies (30–32). The study and the accession number associated with each sample are shown in Table S1, section 3. The sequencing reads were aligned against the human gut database generated by Almeida and colleagues (2) using Bowtie2 and keeping all options to default.

To identify the MAG present in the database corresponding to each AAD-associated bacterial species, we selected the MAGs with ANI values higher than 95 with the NCBI representative genome of each species. The ANI was calculated using the software fastANI (37) keeping all options to default. According to the calculated values, all AAD-associated species have one and only one corresponding MAG in the human gut database.

The comparison between the numbers of species identified in affected patients or healthy individuals was performed using the *Scipy* Python module to perform a *t*-test for independent samples, under the alternative hypothesis that the mean number of present species in healthy individuals is higher than in affected patients.

### Normalized read count metric

To compare the abundances of AAD-associated species in healthy or affected patients, we implemented a normalized read count metric (${Rc}_{n}$), defined as follows:


$$Rc_{n}=\frac{N_{r}\cdot 10^{9}}{G_{l}R_{tot}}$$

where $N_{r}$ is the number of mapping reads on a given genome, $G_{l}$ is the genome length, $R_{tot}$ is the total number of reads in the sample, and 10^9^ is a scaling factor necessary for readability purposes. ${Rc}_{n}$ is, in theory, independent of the number of sequencing reads in a given sample or from the length of the genome for which it is calculated.

To compare the distributions of ${Rc}_{n}$ values of AAD-associated bacteria between healthy and affected individuals, we used the *Scipy* Python module to perform a Mann-Whitney U statistical test, under the alternative hypothesis that the ${Rc}_{n}$ distribution mean for affected patients is greater than for healthy individuals.

## Data Availability

The source code of Metapresence is freely available and can be obtained from the following Github repository: https://github.com/davidesangui/metapresence/ The software is entirely written in Python. It only requires Python3, and the Python modules Pysam (0.19.1 or above) and Numpy (1.23.3 or above). The AAD data sets supporting the conclusion of this article are available at the European Nucleotide Archive with the accession numbers reported in Table S1, section 3.

## SUPPLEMENTAL MATERIAL

Supplemental Figures msystems.00213-24-s0001.docx Fig. S1 to S3.

Table S1 msystems.00213-24-s0002.xlsx Accession numbers of gut sequencing data set and RefSeq genome sequence identifier of various species in simulated communities.

ASM does not own the copyrights to Supplemental Material that may be linked to, or accessed through, an article. The authors have granted ASM a non-exclusive, world-wide license to publish the Supplemental Material files. Please contact the corresponding author directly for reuse.
