# Supplementary material for: Metapresence: a tool for accurate species detection in metagenomics based on the genome-wide distribution of mapping reads
Source: mSystems. 2024 Jul 9;9(8):e00213-24. doi: 10.1128/msystems.00213-24 (PMC11338496; doi:10.1128/msystems.00213-24)
Supplement: Supplemental Figures — Fig. S1 to S3. [file msystems.00213-24-s0001.docx]

**Metapresence: a tool for accurate species detection in metagenomics based on the genome-wide distribution of mapping reads**

Davide Sanguineti^a^, Guido Zampieri^a*^ , Laura Treu^a^, Stefano Campanaro^a*^

a) Department of Biology, University of Padova, Via U. Bassi 58/b, 35121, Padova, Italy

* Correspondence: stefano.campanaro@unipd.it, guido.zampieri@unipd.it

**
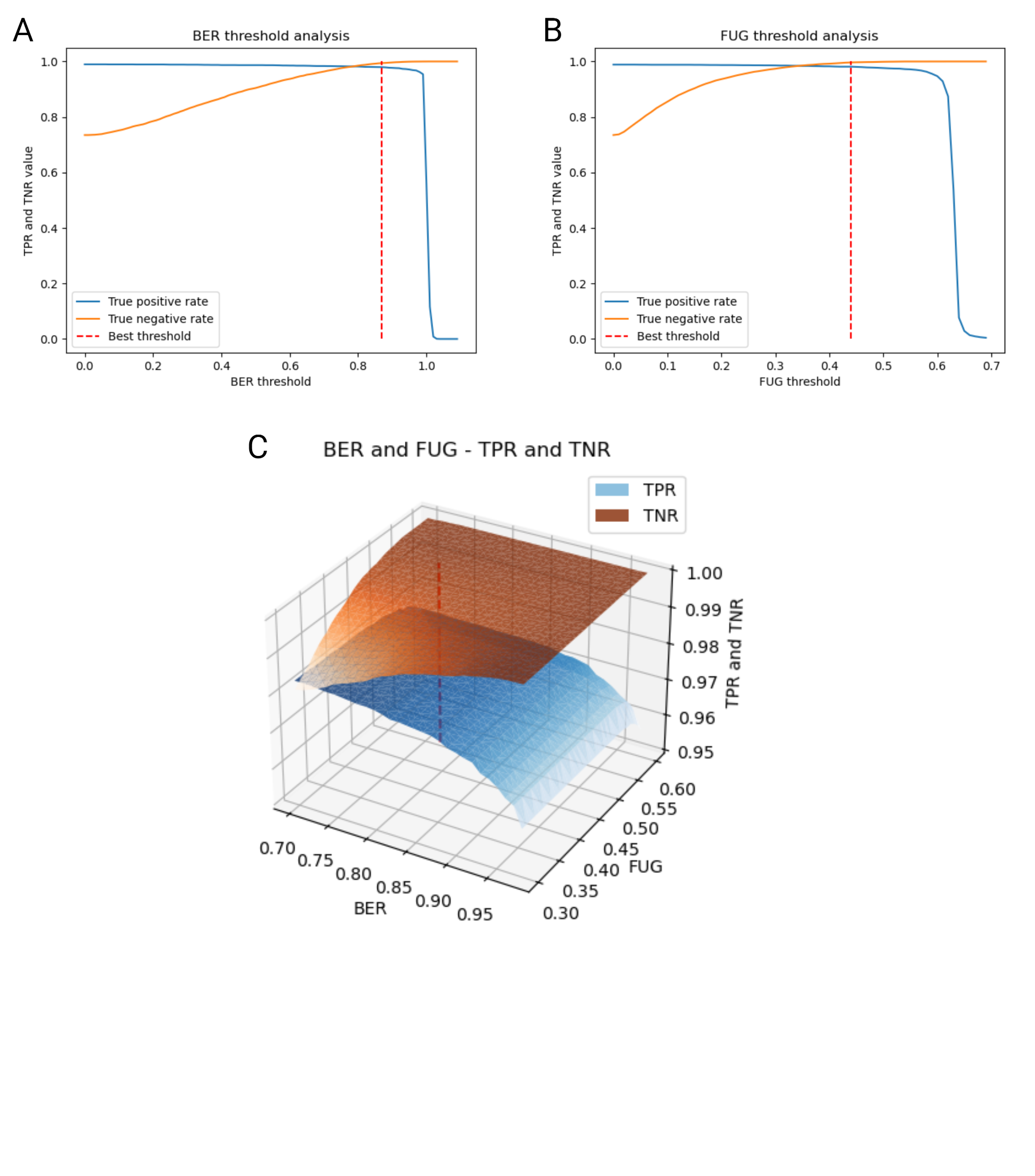
**

**Figure 1**

BER and FUG threshold analysis. In A and B, the x-axis represents the threshold value for either BER or FUG and the y-axis shows the True Positive Rate (TPR) and True Negative Rate (TNR) when using only the corresponding metric to evaluate species presence/absence. The analysis considers ten synthetic communities generated with CAMISIM and described in the manuscript. TPR and TNR refer to the mean value across all the communities. The best value for each metric, identified by the red dashed lines (BER = 0.87, FUG = 0.44), was determined by using the mean between TPR and TNR. In C, TPR and TNR (z-axis) are shown as a function of BER (x-axis) and FUG (y-axis) thresholds when combining them to evaluate species presence/absence. The best combination was found to be 0.77 and 0.50, respectively, and it is shown as a red dashed line.


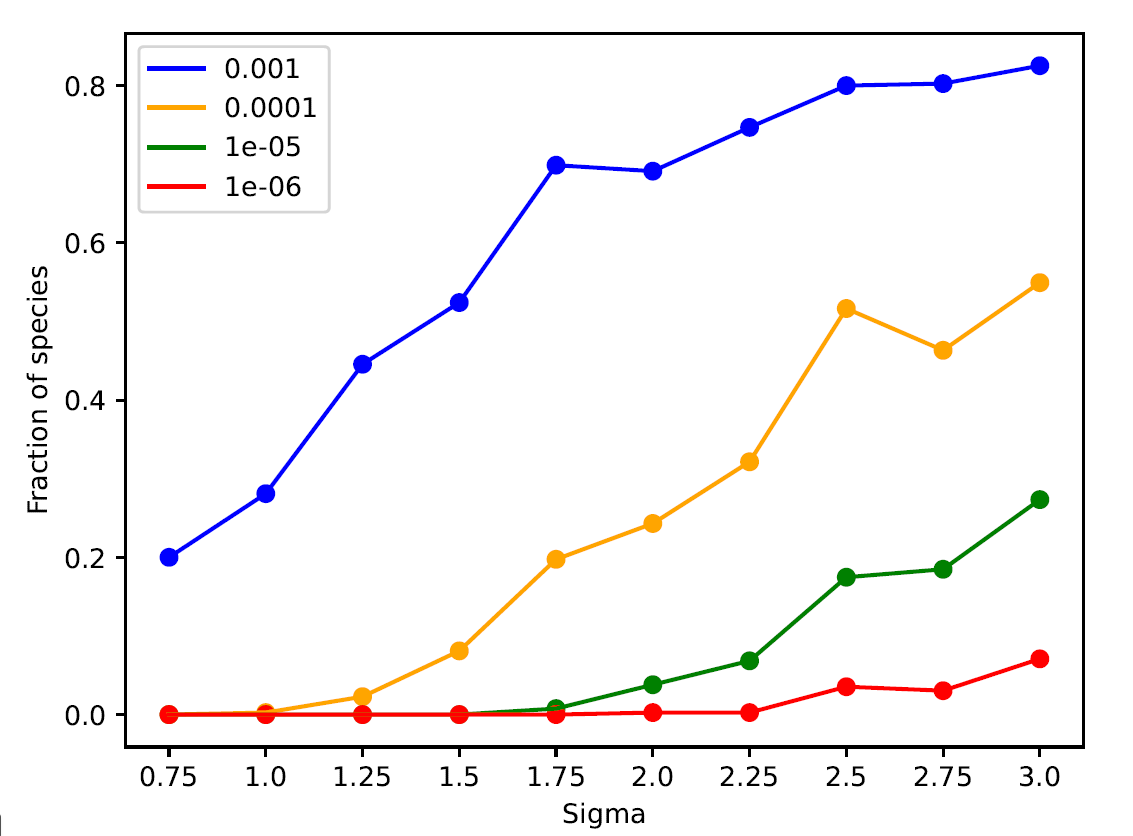


**Figure 2**

Fraction of low-abundant species across simulated communities. Each point on the x-axis represents the 𝜎 value used to generate the abundance distribution in the corresponding simulated community, while the y-axis shows the fraction of species in the community with relative abundance below a given threshold. Each line in the plot corresponds to a threshold as indicated in the figure legend. The higher the 𝜎 value and the higher is the fraction of rare species.


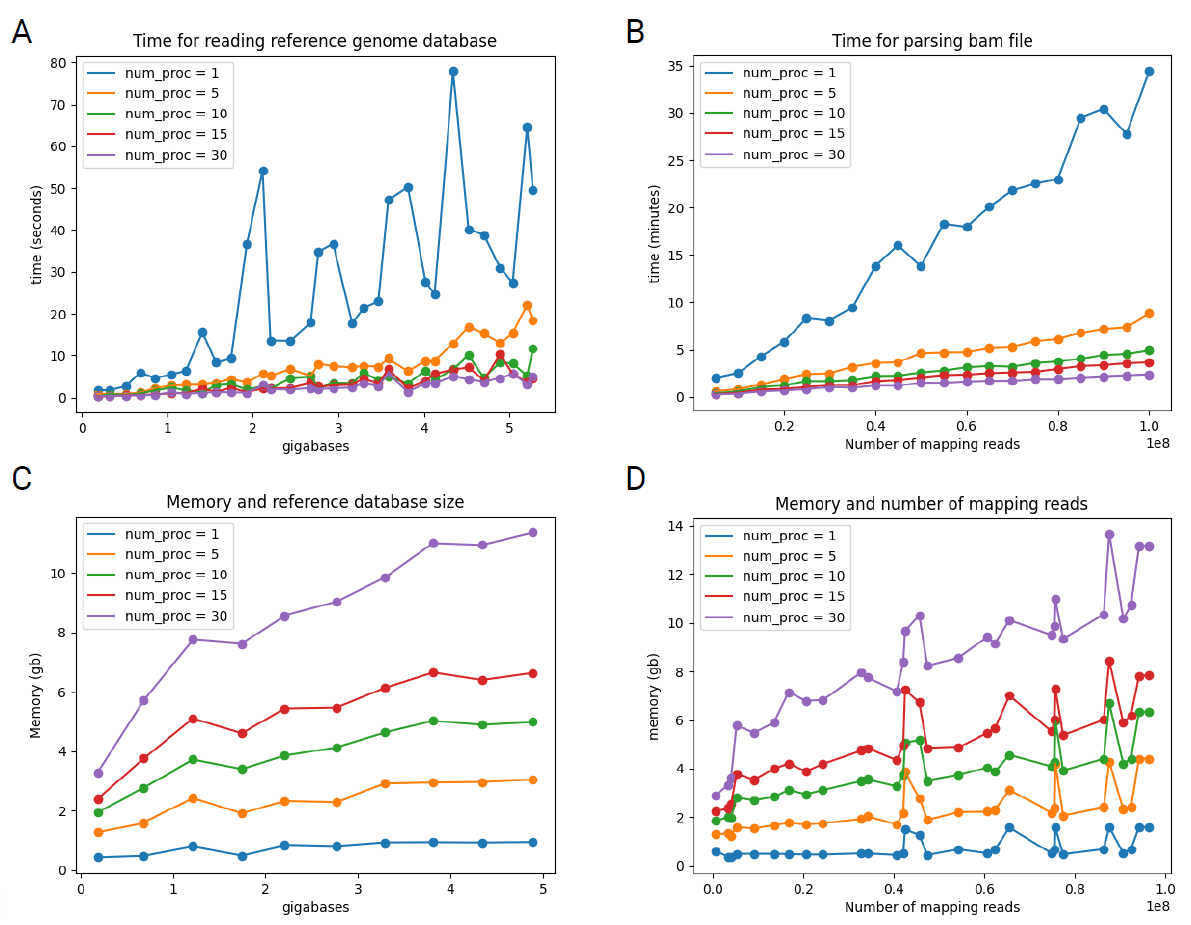


**Figure 3**

Computational performances of Metapresence. Panel A shows the relationship between the time required by Metapresence to parse reference genomes and their total number of bases. Similarly, panel B shows the relationship between the total number of mapping reads and the time required for parsing the input bam file and concurrently calculate the metric values. The relationships are, in both cases, shown for different numbers of processes used for parallelization, as indicated in the figure legends. The total time required by Metapresence is the sum of the times required by these two operations, since the time required for preparing the output files is negligible.

Panels C and D show the dependency between the peak memory of a given Metapresence run and the total size of the reference genomes or the number of mapping reads. Memory usage mainly depends on the number of processes used.
